# Teaching How to Bridge Neuroscience, Society, and Culture

**DOI:** 10.1371/journal.pbio.1001178

**Published:** 2011-10-25

**Authors:** Giovanni Frazzetto

**Affiliations:** 1 BIOS Centre, London School of Economics, London, United Kingdom; 2 European Molecular Biology Laboratory, Monterotondo (Rome), Italy

## Abstract

Societal awareness and transdisciplinarity lie at the heart of a novel educational resource that teaches how to bridge neuroscience, society and culture.

One day when I was still in college, I dared to enter the loftier humanities section, to borrow an unusual book. When the library clerk realized that I was a science student, she seemed taken aback, almost alarmed: Whatever do you need Canguilhem for, if you are studying molecular biology? Don't you have enough to read in your own subject?'

Well, yes, I did. But I figured that the French historian and philosopher of science would help me as I studied the development of the nervous system and principles of clinical neuroscience. Indeed, his thoughts on the boundaries between normality and pathology, and on the role of social norms (see Box 1) in determining what makes a disorder worthy of medical attention, were an enriching addition to the diagnostic manuals. Such adventures outside my own discipline proved instrumental in helping me broaden my views and thoughts and shape future projects.

**Box 1. Definitions**

**Epistemology**

From the Greek “episteme,” epistemology is a branch of philosophical theory concerned with knowledge and how we acquire it. It is connected to notions of truth, evidence, causality, and the justification of models and theories. In simple words, it refers to what constitutes knowledge in a given discipline and to the methods developed to establish that type of knowledge. Every discipline has its own epistemology, based on assumptions and rules. For example, neuroimaging techniques raise epistemological questions as to what is the causal correspondence between the behavioural or cognitive phenomenon under observation and the structural and functional localisation of its component parts.

**Ethnography**

Used in the field of social sciences, especially sociology and anthropology, ethnographic work gathers empirical data that describe specific social contexts and cultures. Within the framework of neuroscience research, it can, for instance, reveal the socio-cultural contexts in which certain phenomena arise—such as a high incidence of anxiety or schizophrenia—and give details about the progression and management of a condition among patients affected by it. Ethnographic work is also used by social scientists to study the research environment of science, as well as the economic, political, and personal factors influencing it (e.g., the research environment of an imaging lab and how knowledge is produced in it). It can involve the use of surveys, archival research, or text and image analysis.

**Phenomenology**

In psychology, phenomenology refers to the study of the subjective lived experience of a state of mind or of a mental disorder. A description of a mental state in phenomenological terms includes personal and narrative details of its symptoms and progress. These are missed by a biological analysis, but they perhaps can strengthen anatomical, hormonal, genetic, or imaging data, especially to match their pathological thresholds with accounts of severe manifestations.

**Social norms**

Broadly speaking, they are agreed-upon, implicit, or explicit rules that shape attitudes, beliefs, values, and complex patterns of social roles, behavioural customs, and interpersonal relationships in a given society and period of time. They can both elicit or limit types of behaviour.

**Subjectivity**

As opposed to objectivity, subjectivity generally refers to our own and unique way of being in the world, experiencing it, and to our ways of thinking, acting, and feeling in relationship with others, both as social subjects and as bodies.

**Transdisciplinarity**

Often distinguished from what one calls inter-, multi-, or pluri-disciplinarity, transdisciplinarity does not simply mean laying two or more disciplines next to each other. It involves the formulation of joint problems within a dialogue among different perspectives and through integration of skills and expertise for their resolution. As has been noted [2], the prefix of the word, trans, is shared with transgressiveness. In fact, transdisciplinarity does not respect disciplinary boundaries and is about transgressing them.

Unfortunately, in most universities, sharp disciplinary and departmental divisions continue to this day and have regrettably translated into the life sciences being taught with scarce attention to their historical and epistemological foundations, or to the socio-cultural, political, and economic factors that influence them.

Likewise, students in the social studies of science, the arts, or humanities are not often directly exposed to how questions in a science laboratory are formulated, or to the design and execution of experiments. Such curricular separation creates a knowledge gap. While this holds true across the life sciences, it is particularly problematic for neuroscience.

Advances in the neurosciences are having a strong and tangible impact on society and individuals and notions of human nature, raising ethical, social, and philosophical questions that range from the value of life and brain activity in vegetative states to the overprescription of psychopharmaceutical drugs, with implications for public health and law. Awareness about these issues and about the influence of one's own research on society is expected of any committed and responsible researcher (let alone the development of skills in communicating scientific findings in a form that can be understood by nonscience experts). And because research in neuroscience addresses broad questions—such as consciousness, mental illness and its treatment, social interaction, emotions, perception, and even aesthetics—that straddle diverse disciplinary territories, we need to give students the tools to understand these issues from diverse perspectives. For instance, mouse genetics, molecular, and cellular biology are apt to identify brain receptors involved in the sensation of pleasure and drug abuse. Neurochemistry characterises their detailed mode of action at synapses. However, addiction is a social phenomenon whose incidence, mechanism, and treatment transcend the laboratory, extending to social dynamics, family histories, environmental risk, and governmental policies. Likewise, even if brain imaging can highlight brain regions at work in hyperactive children, an investigation into whether hyperactivity disorders are primarily of physiological origins or are dependent on culture must go beyond functional anatomy. For all these reasons, a multi-perspective approach to science education is urgently needed.

## Neuroschool: Promoting Societal Awareness and Transdisciplinarity in the Neurosciences

The Neuroschool aims to narrow this gap by providing an international platform to foster societal awareness and transdisciplinary collaborations in neuroscience. The school is open to graduate students and early- to mid-career research fellows in the disciplines of biology, neuroscience, sociology, anthropology, psychology, and history or philosophy of science, and occasionally from the arts or science journalism. About 12 to 15 applicants from around the world are selected on the basis of their academic interests and distinguished research achievements, but also on the basis of a written personal statement in which they express their motivation and genuine aspirations for reciprocal learning. Participants are invited to abandon entrenched positions in their own fields, transgress boundaries in order to unify knowledge, think creatively about their work, and explore together the many known and yet unknown interfaces between neuroscience, society, and culture.

To achieve this goal, each course has a specific theme and runs intensely for one week. It takes place in a neuroscience laboratory so that the nonscience students can experience laboratory life and have a chance to interact with on-site scientists. The classes are taught by experts—both from neuroscience and from the social sciences and humanities—who deliver focused lectures relevant to the theme of the school (e.g., Box 2), most often based on their most recent and compelling work. Before their arrival, participants are given an extensive reading list that equips them with the basic concepts, terms, and arguments of disciplines foreign to their background and, therefore, prepares them to deal with the teaching material. For instance, two schools had psychiatric genetics and brain imaging as their themes. In the former case, the course content reviewed current strategies to measure genetic variation, environmental influences, and behaviour, but also had lectures on how certain kinds of psychiatric disorders arise under given social norms or in certain historical periods, and on sociological concepts of normality and pathology [1]. To cover brain imagery, participants learned about functional brain connectivity and state-of-the-art advanced imaging techniques, but also about issues of data processing, interpretation, and objectivity in science and on the valence and meaning of brain images outside the laboratory and among the public.

**Box 2. Content Example: Psychiatric Genetics: From the Lab to Society and Back**

Behavioural genetics and, in particular, the investigation of psychiatric disorders, have significant societal relevance. Scientific research in this area aims to develop new diagnostic and therapeutic avenues to treat mental illness, but such research has an impact on many aspects of our lives, especially the understanding of disease, normality, subjectivity, and equality.

**Material covered in lectures:**

**About Science**

- Psychiatric nosology (Diagnostics and Statistical Manual entries and disorder classification and symptoms).
- Measures of phenotypes (behaviour rating scales, biological markers, animal models, endophenotypes etc.).
- Gene–environment interaction studies.
- Measures of genetic variation (single nucleotide polymorphisms, copy number genes, genome-wide association studies, etc.).
- Environmental factors, longitudinal studies.
- Principles of epigenetics. How does the environment get under our skin?
- A few specific case studies (depression, ADHD, schizophrenia, anxiety, etc.).

**About Society**

*History and Context*

- When did certain psychopathologies originate and what contributes to their rise in a given time, society, or culture?
- Medicalisation factors (medical community, patients' organisations, media, pharma industry, etc.).

*People*

- Lived experiences of patients with psychopathologies.
- Impact of patients or family organisations on research or health policies.

*Policy*

- Borders of normality/pathology and access to mental healthcare.
- What are the social issues surrounding the use and overprescription of psychopharmaceuticals?

*Social responsibility*

- What is the social responsibility of a neuroscientist?
- What kinds of behaviour are selected for study in the laboratory and why?

**Practicals:**

- Animal behaviour tests
- Histology of rodent brains
- Functional imaging in mice and men
- Human DNA genotyping

**Questions for discussion and input for the “Experiment” (** **Box 3** **):**

- What are the main challenges (epistemic, methodological) in bringing historical and sociological concepts into the laboratory devoted to psychiatric genetics?
- What is the realistic potential in this endeavour?
- What are the most pressing societal questions to address through such a transdisciplinary interaction?
- What is the societal responsibility of a neuroscientist?
- Can and should we use narratives in illustrating the differences in severity, context, and sequence of symptoms?

In addition to lectures, part of the week is devoted to practicals, which are usually hands-on laboratory tasks (e.g., DNA genotyping, animal behavioural tests, microscopy, or imaging data acquisition and analysis) to help the nonscientists become acquainted with routine experimentation. Methodological workshops are also run, but this time by the social-science or humanities students, who show the scientists the techniques they use to study society and culture or to analyse how science research is conducted (e.g., ethnography, surveys, interviews, archival research, or text and image analysis). Participants also give short presentations on their own work and receive feedback from one another and from the expert faculty present. Ample time is dedicated to global discussions that often continue during social and recreational activities at the end of each day.

In addition to exchanging ideas and concepts and learning different techniques and methods, a central component of the course is the “Experiment,” a specific exercise to train the participants to design and solve a specific research question (Box 3). This exercise illustrates why addressing some questions can benefit from an encounter between experimental science and social theory, or input from the arts or other fields within the humanities, such as philosophy. For instance, suppose that we are interested in explaining the heterogeneity of manifestations of anxiety. While we measure genetic variation and utilize brain imaging to pinpoint functional differences across individuals, an approach that could be shared by psychiatrists, epidemiologists, and geneticists would investigate the variety of methods used to identify pathology (including psychiatric categories, psychometric instruments, or biological markers). However, ethnographic work by sociologists or anthropologists would reveal the socio-cultural context where the incidence of anxiety is higher and provide information about how the condition progresses or about types of individualised treatments, and prompt further investigation of biological variation in severe cases.

**Box 3. The “Experiment.”**

At the start of the week, participants are divided into groups reflecting their mix of disciplinary backgrounds and tasked with designing an original research project, which, while remaining scientifically rigorous, breaks down conventional disciplinary barriers. This process serves at least two main purposes. First, it makes them think about an appropriate research question to solve, one that has pressing societal relevance but is also amenable to transdisciplinary inquiry [2],[3]. It encourages participants to identify the main gaps, contradictions, or missing links in their shared fields of interest. Second, it helps them identify the most suitable techniques and collaborations that will enable them to answer their question most successfully.

Although they are asked to imagine they have all financial resources at their disposal, participants have to justify the chosen methodologies. Each of them acts as a gatekeeper to ensure rigour in the specific set of methodologies used in their own discipline.

On the last day, participants prepare a short presentation to introduce their work to the group. The presented projects enter a competition and, following a discussion during which groups comment on each other's projects, the faculty selects the winning group on the basis of the following criteria:

1. Originality and daringness: the project must pace unexplored, or unresolved, territories of knowledge and experimentation.
2. Feasibility: the research can stretch in time and resources, but it must ensure its feasible realisation within a concrete time framework.
3. Transdisciplinarity: participants need to ensure that they understand and employ each other's languages and that the chosen methodologies are effectively integrated and justified.
4. Communication: the aims and modalities of the research proposed must be clearly outlined in a language comprehensible to everyone in the group.

The winning group receives a prize and, where possible, is invited to conduct the experiment planned in one of the faculty members' laboratory.

During this exercise, the students learn how different disciplines can help each other and to what extent their methodologies can be used complementarily. They appreciate the freedom of developing a research agenda that is conceptualised as transdisciplinary from its onset. They also recognise how challenging it is to merge two seemingly different worlds of knowledge. The challenge is primarily epistemological, in that different epistemological practices with traditionally distinct methodologies and approaches need to coexist within one research endeavour (please refer to Text S1 to read a dialogue among Neuroschool alumni who reflect on their participation in the “Experiment”).

Rather than simply deliver basic material or exchange data and insights, therefore, the school provides an opportunity for a group of highly motivated scholars to identify their shared, nonresolved questions, integrate concepts and methodologies across different disciplines, and delineate the most suitable research modalities of research to address them.

## Output and Resonance

There have been four Neuroschools since 2008 comprising 60 talented and enthusiastic individuals. The goal has been to provide a unique and unconventional teaching platform in which scholars can maintain their own scholarly identity, while questioning their own and one another's disciplinary assumptions and limitations.

The material and activities covered during the course remind the nonscientists that laboratory experimentation imposes operational boundaries and protocols that need to be respected to produce evidence-based knowledge. However, they also show the science group that the choice of one method over the other can bear enormous societal relevance—for instance, choosing one diagnostic method or behavioural measure over another affects the inclusion criteria for epidemiological studies and decisions for pharmacological treatment; publishing the brain mapping of an experimental paradigm testing social cooperation can swiftly generate a popular belief that such a complex human interaction is only a matter of genes and neuronal circuits.

The Neuroschool has been successful in inspiring participants to disseminate the material and messages learnt both within their community and more widely. Some alumni have already put their new knowledge and way of thinking to use, ranging from the joint organization of conferences, the creation of novel teaching material, the coauthorship of articles, and research collaborations [4],[5]. The winners of the Experiment competition (Box 2) among the 2009 alumni group continued their project at the University of Aarhus, Denmark.

All the scholars who participated told me they wished they had received a Neuroschool-like type of tuition in their home departments. They regarded the week spent together as precious time in which it was possible to find like-minded colleagues with whom to take a step back from their work, look reflectively and critically on it, and explore creatively how it might be done otherwise. They also emphasized mutual trust and respect for one another's positions as essential for the establishment of a fruitful dialogue.

As has been noted elsewhere, one virtue needed in transdisciplinarity is patience, and in high amounts [2]. It takes patience and commitment to understand and adequately employ the language and methods of other disciplines. Introducing context or adding a social or cultural dimension in a laboratory experiment increases complexity and involves time. Beyond the tangible and immediate output cited above, the tuition offered at the Neuroschool is a long-term investment that we hope will impact future research and promote innovation in the field of neuroscience. The history of science shows us that it is precisely this type of heterologous knowledge at the interface between disciplines, rather than at the centre of well-defined areas of knowledge, where great discoveries and surprising results are more likely to occur (think, for instance, about the discovery of the structure of DNA).

This is surely a challenging and uncertain approach, but it is also intellectually very stimulating and rewarding. As one Neuroschool alumna told me at the end of a course: “Transdisciplinarity leads us to ask questions—and in a language—we may never have known existed.”

## Future Perspectives

The motivation and rationale behind the Neuroschool are simple. Neuroscience is inexorably enmeshed within the society and culture we live in and scientists should have the opportunity to investigate the implications of this relationship [6]. Yet, the past tradition of educational programmes has been such that the more senior generation of scholars still finds it difficult to engage in concrete dialogue with other disciplines. They are simply not used to it because no one taught them how to. This could be reversed if we invested in changing the way we educated the emerging generation, by immersing them early on and repeatedly in their career into a broad and transdisciplinary mode of learning.

The Neuroschool model can be applied to exploring various topics in neuroscience with societal relevance—such as empathy, volition, addiction, psychopharmacology, etc.—and could easily be extended to the many other scientific disciplines that have societal relevance. Such an approach could become an integral part of the current science curriculum, both at the undergraduate and postgraduate level, by introducing broad courses that bring lecturers and students with different backgrounds and aspirations to tackle complex and global issues that link science and society. The motivation for such a programme is not merely intellectual. Educating students differently is important to ensure the formation of socially responsible global citizens who could make a meaningful contribution to health, science, and public policy. We are used to assessing academic quality within boundaries of distinct disciplinary structures, while transdisciplinarity is precisely about transgressing them. It is important to create suitable institutional spaces for the selection, assessment, and delivery of such emerging transdisciplinary aspirations—such as in journals, research institutions, funding organisations, and evaluation committees.

Transdisciplinarity would not be seen as a threat if we collectively supported it as one of the accepted ways to produce science and if we embraced the risks and uncertainties connected with it. Venturing into other sections of the library would no longer be an unusual occurrence and would be appropriately encouraged and rewarded.

I tell all the Neuroschool participants that they are rebels, constructive rebels. Freeman Dyson reminds us that “there is no contradiction between a rebellious spirit and an uncompromising pursuit of excellence” [7]. Constructive rebels bring a draft of fresh air and make an impact without overlooking rigour and brilliance.

## Supporting Information

Text S1 **Becoming transdisciplinary? Three dialogues.**
(DOC)
