# Supplementary material for: Teaching How to Bridge Neuroscience, Society, and Culture
Source: PLoS Biol. 2011 Oct 25;9(10):e1001178. doi: 10.1371/journal.pbio.1001178 (PMC3201915; doi:10.1371/journal.pbio.1001178)
Supplement: Text S1 — Becoming transdisciplinary? Three dialogues. (DOC) [file pbio.1001178.s001.doc]

**Becoming Transdisciplinary? Three Dialogues**

Morten Hillgaard Bülow, Udi Butler, Lindsay Chura, Alison Cool, Thomas Dresler, Marie-Louise Holm, Jenell Johnson, Neely Myers, Samantha Ranking, Daniel Stjepanovic, Louise Whiteley.

The Neuroschool, and in particular the Experiment contest with which the week culminated, was a transformative experience for the 2010 Alumni. The participants were determined to continue reflecting on the ways in which this experience had affected their perspectives on research and so, scattered across the globe, each Experiment team organised a Skype meeting to discuss a list of questions derived in collaboration with Dr Giovanni Frazzetto. Their aim was to produce group dialogues that would help participants clarify what they had learned about transdisciplinarity, and which would also communicate to others the experience of taking part in The Experiment. Further, the group production of this report was itself a way in which to continue addressing the challenges of transdisciplinary collaboration.

**Group 1**

The first group was comprised of neuroscientist Lindsay Chura, pursuing structural and functional neuroimaging research into autism spectrum disorder; neuroscientist Samantha Rankin, researching neural connectivity patterns in ADHD; Louise Whiteley, a researcher in neuroethics and science communication with a background in theoretical neuroscience; and social anthropologist Udi Butler interested in the methods of neuroscience, especially the relationship of first and third person perspectives.

**The Experiment**

The title of the collaboratively designed experiment was ‘Stress and experience through the diagnostic process: Phenomenological, symptomatic, and biological measures’. The experiment sought to shed light on fluctuations in patient stress levels within the context of lived experience during the diagnostic process, and asked whether these diverse measures would differ for Psychiatric versus Neurological conditions and by severity of disorder. The group’s strategy for integrating their various interests, perspectives, and experiences was thus to choose a particular event or process – in this case, diagnosis - and then attempt to approach it through a combination of different methodological lenses that was more than the sum of its parts. For example, the group was interested in whether adding phenomenological reports, taken over a period of time before and after diagnosis, might deepen clinicians’ insight into the patient’s experience and how these reports might be shaped by the person’s attitude towards the “biological basis” of their disorder.

***What were your expectations or ideas about the experiment beforehand?***

**Samantha:** I wasn’t certain what was meant by transdisciplinary work – was it people from different disciplines working on one project, or working on different aspects of the same question?

**Louise:** Maybe it was good not to know what exactly the rules of the game were! I remember when we were discussing our study it took us ages to let go of the idea of integrating brain scans; were they relevant or did we want to include them as ‘proper neuroscience’ data?

**Udi:** Yes, I think we began hung up on techniques rather than looking at the question itself, and we really started getting somewhere when we focused on the questions that interested us.

**Louise:** Though some questions didn’t seem to lead us to transdisciplinarity I think…

**Udi:** I guess it’s more about realms of interest or possible inquiry: like we were interested in diagnosis, changes in experience, phenomenology – that’s when we began to find a common ground between the disciplines of how you could have something that tried to engage with these different elements.

**Samantha:** That was important because trying to make this transdisciplinary experiment without having an idea of what we’re trying to achieve through the experience was difficult; we realized that the end goal of trying to do something in a transdisciplinary manner wasn’t the right starting point.

**Louise:** And also I think that until we tried to figure out what an experiment would be in practice we weren’t clear what the difficulties would be, what the problems would be.

**Samantha:** Yeah, we’d been discussing these questions since the start of the week but they really started to make sense when we tried to design the experiment.

**Louise:** An important shift for me I think was the idea that it doesn’t have to be perfect, there isn’t going to be only one chance – we should just try and work through something together and see where it took us. I didn’t feel pressure for the experiment to demonstrate what was most of interest to me. I guess in the ‘real world’ there’s more pressure – it’s more like there’s only one experiment, the stakes are higher.

***Was there anything you were reluctant to compromise on at the beginning?***

**Udi:** The four of us were quite open, and didn’t seem to have any disciplinary hang-ups as such, I didn’t feel that I was protecting anything in that sense.

**Louise:** Did you expect you might need to beforehand? Lindsay and Sam, you were more from a neuroscience background, did you feel you would have to defend experiments?

**Samantha:** No. I have a bit of social science in my background too, and never felt I was going to have to defend science. I think it made it easier because our group had a good mix in terms of understanding scientific methods and constraints, even if we weren’t all scientists.

**Lindsay:** I think the larger point here to consider is the fact that while we are all pursuing different areas of research, it’s our shared interest in the field of neuroscience as a whole that brought us together and the different lenses through which we view neuroscience helped to inform rather than hinder the discussion. That’s what’s unique about the NeuroSchool experience.

**Samantha:** Louise, you were talking about learning about the limits of some imaging techniques from me and Lindsay. I also remember asking Udi what kind of things from anthropology would you bring in in terms of methods; trying to get from other people what would be interesting to them…

**Udi:** I was at a conference a few weeks ago entitled ‘What is it with the Brain these days?’, and one of the most interesting questions in the field of the social studies of science which seems to be dividing people is that there are those who take a critical approach of say neuroscience and then there is the approach, exemplified by the Neuroschool, which raises the possibility for an engagement between the social and natural sciences. A ‘productive engagement’, even if also critical, rather than a divisive critique.

**Louise:** I agree, and it’s very difficult to live in that place... I think a really valuable thing about Neuroschool was exposing everyone to the labs, to the complexity and skill and beauty of neuroscience as well as its critique, to try and understand what each other’s work is like...

**Samantha:** …and in terms of having been together throughout the week, I think it would have been much harder to have the conversation we had if we hadn’t already gone through shared experiences like visiting the labs and seeing how people thought differently about the same experiments.

**Lindsay:** Yes, a sense of collegiality had really been fostered through the different activities we all participated in, we’d already established a great working dynamic.

**Udi:** Two key concepts I came away with in thinking about transdisciplinarity and collaboration more broadly are trust and friendship – it really made me think about how much of that creative and collaborative potential can only happen in spaces where there is some level of friendship and trust, not a sense of insecurity about having to defend or hangups about the unknown other.

**Samantha:** I think the presentations of your work or research interests at the start of the week was really important to that – talk about why you do it, give a sense of your interests.

**Louise:** Yeah, giving people a sense of why you care about the work you do, why you think it’s important – and that might be on a personal level as well as wanting to address an important problem – makes it harder to see people purely in a critical or defensive way.

***What do you think worked well about your experiment?***

**Udi:** I think one thing that was successful about our experiment was that although we had quite a rigorous scientific method and approach there was an openness to themes that might not be part of mainstream science, like the idea of phenomenology or experience as being something worthy of investigation, and ideas about diagnosis and how it feels – an embrace of something that is not mainstream scientific questions.

**Louise:** But I think the questions we looked at also made it more possible than it might have been to take this kind of approach… I’ve been reading about why Foucault chose to work on Psychiatry and on crime and punishment – the idea that the intricacies of how particular scientific discourses foster particular ways of thinking about ourselves would be easier to explore in a more concrete science like Psychiatry as opposed to Astrophysics, say, where it’s much harder to challenge the epistemology…

**Udi:** Mm, so I’m wondering, thinking about other areas in neuroscience, looking at say vision, whether it would be much harder to do this there?

**Louise:** The concepts seem less up for debate I think, even though many of the same questions would apply about who defines what’s important about vision, how and where we study it, why…

***What would you do differently next time?***

**Samantha:** I think I’d be more aware of the statistics, the limitation from proposing so many measures to be compared that actually made the analysis we proposed quite unrealistic! I was quite happy with the way ours worked out apart from that… Though I guess it was quite weak on the neuroscience side, we didn’t understand the biological technique we proposed very well.

**Lindsay:** What other kind of measure do you think we could have used?

**Samantha:** That’s the problem, I still can’t think of anything!

**Louise:** And in a sense we made it easier for ourselves by letting the biological play a smaller role, though that came out of following the question we asked, not on purpose. Also, we seemed to be missing a science studies element – research into how the scientific practice itself was working – was that a good thing? Udi, you were talking about that earlier?

**Udi:** Yes, but I do think there was a sense of taking a critique of diagnosis having an effect – using an experiment to test out a social science assumption; both a constructive and an experimental critique I guess.

**Louise:** I love that way of looking at it.

**Group 2**

The second group consisted of Marie-Louise Holm, a historian and philosopher researching norms and values in medical research; Neely Myers, a medical anthropologist who conducts research on culture, stress and mental health as well as the understanding and treatment of people diagnosed with schizophrenia; Thomas Dresler, a psychologist working on neuroscientific and psychiatric research; and a sociologist (unnamed because they did not participate in this conversation) researching emotion and politics in relation to organ donations.

**The Experiment**

The experiment of the second group examined how gene-environment-interaction (gene(s) affecting the serotonergic system and the attachment style evident between parent and child) affect later behavior and brain activation. While this experiment was carried out, three research projects from a historical/philosophical, a sociological, and an anthropological point of view would critically examine the premises of the gene-environment experiment and the general tradition of studying attachment within psychological research.

***How did you choose your experiment?***

**Thomas:** We did not really have a common question, but had to negotiate on finding one while keeping in mind that it should be approached from our different perspectives. So it was simply the fortuitous combination of disciplines that made us choose – not really the aim of the experiment – but rather the approach of it.

**Marie-Louise:** I think the situation of being a group of researchers that was formed randomly and then having to decide on a common experiment was not a very realistic setting for the group-work. In the interdisciplinary group-work I have done before you usually form a group because you have a common interest in a topic/problem.

**Neely:** We were all focused on obtaining data that we each thought would be interesting to work with, and so in the end, our experiment did not seem collaborative. We ended up having a core psychological experiment which the social scientists would analyze, and the social science projects seemed like an added layer. In the end, it seemed that we failed to think creatively in new ways beyond our usual methodologies to truly weave a new kind of cloth rather than making a patchwork quilt.

***Was societal relevance important to you?***

**Thomas:** Of course. We all suggested different experimental topics concerning questions of societal relevance. It was essential to us that the question should aim at important issues such as gene-environment interactions on personality and disorder to elucidate processes of disorder-relevant development. However, in my view, we did not really succeed in the end.

**Neely:** Yes and no, I would say. We chose the psychological project about attachment, because this question had not been settled yet. But it was not because we found it to be the most societally pressing question to be solved, but more that we had to move forward in the process because we had run out of time. We wanted to do something societally relevant, but I think more importantly we were trying to do something academically relevant to our specific fields and publishing venues. Relevance to society may have been a better place to begin.

**Marie-Louise:** I think that everyone was actually very concerned about societal problems in their research area. But in this situation the struggles within the group with collectively finding a common research question and design shifted the focus to defending the relevance and legitimacy of our specific disciplines. This often happens in group-work. We did not find a common point of departure, and so a feeling of understanding each other and collaborating on the same project that has amazingly synergetic effects, and leads to creative new ways of approaching and analyzing a problem did not really happen for us.

***What were your main struggles?***

**Thomas:** Communication across disciplines was not a big problem as we were able to make ourselves clear. I think a main struggle was methodological issues, which differed for our disciplines. It was also hard to find a research question appropriate for all participants. We definitely had too little time to make clear and choose the very basis of our experiment: the concepts, methods, approach, etc.

**Marie-Louise:** In my view a main problem was how the project was designed in terms of which aspects of the project were emphasized most. Because the psychological aspect carried most weight in the original formulation of the project, and the parts of the analysis using sociological approaches were added to, but not integrated in it, they did not seem essential or indispensable. We had problems as a group with re-thinking and re-formulating the project in a way so that the relevance of different sociological aspects of the project became clear.

**Neely:** I think the social scientists were maybe more trained in understanding the language and methods of the neuroscientists than in reverse. A major part of the NeuroSchool lectures focused more on explaining neuroscience than social science. In our group, we had different understandings of how you can design a meaningful project, what counts as data, which questions you can ask, and why you want to ask them. At times, I felt that this reproduced a traditional hierarchy of knowledge where results of “hard science” counted more than “soft” social science approaches.

**Thomas:** It was a general problem that we did not develop the experiment collectively from the beginning. Actually, it would probably have been better if none of us had planned anything before we met if we were to really engage in interdisciplinary creative thinking. I think we could have designed a better project if everybody had had the chance to start at the same point and work together on a fresh idea.

***What were the key sacrifices group members needed to make?***

**Thomas:** First we tried to come up with a totally new idea and create a project together. At that point I wanted to give up all of my proposed ideas as a new idea developed by all of the group members would be the best. However, this did not really seem to work out, mostly because of the time pressure, and in the end I pushed for us to make a decision.

**Marie-Louise:** Because of the time pressure I had to give up explaining my ideas for how to make the project more coherent and balanced. One of my ideas was that the design of the psychological experiment could benefit from a working process where a psychologist, an anthropologist, a sociologist, and a historian/philosopher at different stages in the research process collectively discussed the premises for the experiment and its possible consequences for patients and society. However, I think I failed to explain the relevance of this approach to the others.

**Neely:** I felt I compromised a lot for our experiment, which I thought was still not particularly feasible nor collaborative. Maybe the presence of a strong transdisciplinary mentor or some clear directions on and good examples of how to actually be transdisciplinary would have helped. When I saw the other group’s experiments, I realized that none of us had ceded any of our disciplinary territory to each other as in the other groups. We needed more overlap.

***What was in your opinion the successful 'transdisciplinary' element in your experiment, and how did 'feasibility' affect your choices?***

**Thomas:** I liked the way we were really talking about a time schedule and experimental feasibility for our experiment.

**Marie-Louise:** Feasibility affected much of our discussion about the design of the project – too much, actually, I think. In transdisciplinary group-work it is very important to have an initial phase where you don't think so much about feasibility, but develop wild ideas and explore different possibilities of combining the expertise of the group members, play with ideas of different approaches, and generally explore which kinds of interesting questions you can detect in the topic you’re working with. Later you must approach the project in a more realistic way and decide how you can make it feasible, abandon some of your ideas, and make it as focused and coherent as possible. The premise that we had to formulate the experiment in a few hours speeded up the process of reflecting on the project in a way that made us think in too narrow circles.

**Neely:** I agree with Thomas. Feasibility is just a reality in research that you actually want to complete! But I also agree with Marie-Louise. Somehow our thinking became too narrow, which stymied our productivity and left us feeling deflated. These are all great lessons for our next attempt at being transdisciplinary, though!

**Group 3**

The third group consisted of Jenell Johnson, a rhetorician researching the epistemological and communication issues involved in the formation of 'neuro-disciplines'; Morten Hillgaard Bülow, a historian and philosopher of science currently investigating the history of the concept of successful ageing within ageing-research; Daniel Stjepanovic, a neuroscientist investigating the effects attention has on the brain's ability to process emotion, and what effect genetics has on this relationship; and Alison Cool, an anthropologist currently investigating the use of biological and genetic models in contemporary behavioral economics based on ethnographic fieldwork in Sweden.

**The Winning Experiment**

After suspending disciplinarity for a week, team 3 agreed that it was disciplinarity itself that they wanted to explore for the experiment contest. The idea for the experiment was initially inspired by a classic problem in game theory, the "Prisoner's Dilemma," which has been taken up by behavioral economists to investigate the role of preferences for cooperation and altruism in strategic decision-making scenarios. Alison, who proposed the idea, initially was interested in utilizing the dilemma as a common setting where researchers from several disciplines could each draw useful data.

Drawing on previous neuroeconomic studies looking at the neural basis of altruistic punishment, researchers could use PET to look for possible changes in the activation of the relevant part of the brain, the dorsal striatum, of the research subjects while they choose a strategy in experimental games described in different ways. This, Alison thought, might take previous work exploring the role of these situational construals in the laboratory context one step further, and might show how research subjects respond to experimental protocols at a neural level. Furthermore, qualitative exit interviews with research subjects would help to understand how decisions in the experimental context were possibly understood differently by the experimenters and the experimental subjects. As such, the experiment would equally appeal to a neuroscientist, an economist, and an anthropologist, who would each come away with their own sets of data.

As the group discussed the idea, a much more interesting question began to emerge. What if the point was not the experiment itself, but the differences and commonalities in the kinds of data collected? What if the experiment could be used as a way to investigate cooperation between the disciplines that would go beyond separate strings of data collection? In other words, Prisoner’s Dilemma would not be the aim of the experiment. Instead, it was an arbitrary occasion to facilitate dialogue and exchange between disciplines. The group therefore proposed that before, during and after the experiment, researchers would share their hypotheses, data, and conclusions in order to identify points of productive friction between them: dialogue was not just a means to an end, but one of the ends of the experiment. Our experiment was thus both multidisciplinary, in that each researcher could bring something back to her home field, and transdisciplinary, in that each researcher sought to go beyond discipline in order to gain novel perspectives on his or her subject matter and to ask new and better questions, including broader questions about human behavior and the nature of inquiry itself.

***What do you think made your experiment work?***

**Jenell:** I think that what made our experiment work was that first and foremost we wanted to develop a project that equally valued one another’s input and disciplinary perspective.

**Morten:** I totally agree. I think this may be a premise for successful transdisciplinary teamwork in other situations as well. Looking back on it, what made our experiment win, I think, was that we didn't try to apply some disciplines 'onto' another, but that we kept them equal, trying to find a way to do the experiment so that it would be a gain for everyone. The experiment was not meant to be neuroscience, it was meant to be transdisciplinary; that is, more than 'just' neuroscience or any other discipline in itself.

**Daniel:** In essence, the primary goal of our experiment was to ensure that none of us had to sacrifice his or her interests.

**Alison:** Yes, but the question of perspective was also central, as we imagined the varied outlooks and intentions of our scientific actors. By allowing a single event – a research subject choosing a strategy in a prisoner’s dilemma game – to be interpreted through the methodological lenses of multiple disciplines, we hoped to encourage the multiple perspectives that had proven so fruitful for us during Neuroschool.

**Daniel:** True. For me, both the experiment and the Neuroschool in general allowed me to observe how other disciplines view the scientific method, as well as providing insight (even hands-on experience) with other forms of inquiry.

**Morten:** Yes, it was a unique experience...

**Jenell:** I definitely agree. Although I’ve been working in science studies for a long time, I’ve had surprisingly little experience working with scientists – and certainly never had the opportunity to spend time in a lab. This made Neuroschool one of the most intense scholarly experiences of my lifetime.

**Alison:** Me too! It is rare to have the opportunity to put theories about interdisciplinarity into practice. For this reason, having the chance to collaborate with a philosopher, a rhetorician, and a neuroscientist to design an experiment was a transformative experience. I believe that being invited to witness the day-to-day work lives of the researchers in Würzburg--and hearing the insightful and sometimes surprising questions of our fellow visitors--helped us to think deeply about the practical demands of scientific labor.

**Morten:** Yes, being in the room where this happens makes you think about and understand neuroscience much better than reading about it. I guess being exposed to each other's research environment – the materiality of it, even if it consists mostly of a messy desk filled with piles of books and articles for some of us – help your understanding immensely. This understanding was also necessary for cooperating on the Experiment, I think.

**Jenell:** It’s one thing to read about fMRI. It’s another thing to stand on top of a giant humming magnet after having taken off your jewelry for safety’s sake. It’s one thing to read about translation theory. It’s another thing to watch a slumbering mouse.

**Alison:** Yes, like when we saw a paper towel folded into a pillow for a mouse on her way into an MRI, a graduate student carefully tracing the paths of neural connections on a computer screen to create a readable image, and other, similar scenes of attentiveness and intimacy.

**Jenell:** And, most poignantly for me, it’s one thing to read about neuroscientists and how they develop theories and experiments. It’s another thing to get to know them, to appreciate how their minds (brains?) work, to listen to the questions that they have, to share bottles of wine with them, and to call them friends.

**Daniel:** I'm seeing a trend here, every few minutes our discussion descends into flattery. And I like it.

**Alison:** Maybe this is part of our method: lots of compliments.

**Morten:** Yeah, complimenting each other seems to be an essential part of making interdisciplinarity work! There is a need to build up trust, which both our democratic approach to selecting the experiment and the compliments – and the wine tasting during the week – helped do.

***Did you have to sacrifice anything?***

**Daniel:** I think there is often a tendency amongst scientists to dismiss, or at the very least ignore, other forms of inquiry. This is a dangerous position as it ignores many facets of the human experience that are outside the scope of scientific inquiry. Questions that are fully within the scope of the scientific method are likely to benefit from a transdisciplinary approach, as a deeper understanding of a phenomenon will always be more conductive to the truth than a shallow or narrow understanding. Would you guys say there is a fear in the social sciences that interdisciplinary research means giving up something that makes you a social scientist or humanist?

**Morten:** Good question - I think some people might think that…

**Alison:** Maybe there's a fear that you will be forced to give up your tools of critique, that you will have to give up the insider-outsider perspective of anthropology and just be on the inside.

**Jenell:** Or the fear that you’re seen as a commentator or consultant on the ‘primary’ natural science project, and your research isn’t valued for its own sake.

**Morten:** Is it different in your discipline, Daniel?

**Daniel:** I don't think so, it was sort of the attitude I had before the experiment; that crossing disciplinary boundaries means, as Alison said, giving up your tools. For me it is being able to break something down to this small, very controlled question - which isn't what an anthropologist, historian, philosopher or rhetorician might be interested in doing.

**Morten:** That is an important point.

**Alison:** Yeah! We want to keep as much complexity as possible.

**Daniel:** Something that became obvious during the NeuroSchool is that my definition of transdisciplinarity required revision. My initial understanding of the term was biased strongly by a neuroscience perspective. The term ‘transdisciplinary’ evoked the combination of closely related fields within biology such as the combination of genetics with neuroimaging to attempt to understand the molecular basis of a particular neural signature, cognitive process or behaviour. But if transdisciplinarity only ever encompasses highly similar fields, there is possibly less to be gained than through the combination of more distant disciplines. True curiosity about the world cannot be the exclusive domain of a single discipline.

**Morten:** I think we found that transdisciplinarity is really about getting something, not giving something up. In other words, our different perspectives have to be viewed as resources, not obstacles.

**Becoming Interdisciplinary: A Conclusion**

In many ways, the Experiment contest was a microcosm of our week in Würzburg. We discovered that the sparks that result from disciplinary friction can result in a productive burn of inspiration that has led us to actively seek collaboration with those outside our home fields, who we discovered were not epistemological adversaries but scholarly friends, differently-timbered voices in dialogue.
